# Unveiling the clinical spectrum of relapsing polychondritis: insights into its pathogenesis, novel monogenic causes, and therapeutic strategies

**DOI:** 10.1186/s42358-024-00365-z

**Published:** 2024-04-16

**Authors:** Blanca E R G Bica, Alexandre Wagner S de Souza, Ivânio Alves Pereira

**Affiliations:** 1 Reumatology Division of Hospital Universitário Clementino Fraga Filho (HUCFF), Universidade Federal do Rio de Janeiro, Rua Esteves Junior 62, CEP 22231-160 Laranjeiras, Rio de Janeiro, RJ Brazil; 2 Rheumatology Division, Escola Paulista de Medicina, Universidade Federal de São Paulo, São Paulo, Brazil; 3 Reumatologia da Universidade do Sul de Santa Catarina-UNISUL, Florian?polis, RJ Brazil

**Keywords:** Relapsing polychondritis, Cartilage, Auricular chondritis, Nasal chondritis, *UBA1* gene, Somatic mutations, VEXAS, Ubiquitin, Myelodysplasia, Autoinflammatory disease

## Abstract

Relapsing polychondritis is a rare multisystem disease involving cartilaginous and proteoglycan-rich structures. The diagnosis of this disease is mainly suggested by the presence of flares of inflammation of the cartilage, particularly in the ears, nose or respiratory tract, and more rarely, in the presence of other manifestations. The spectrum of clinical presentations may vary from intermittent episodes of painful and often disfiguring auricular and nasal chondritis to an occasional organ or even life-threatening manifestations such as lower airway collapse. There is a lack of awareness about this disease is mainly due to its rarity. In 2020, VEXAS (vacuoles, E1 enzyme, X-linked, autoinflammatory, somatic) syndrome, a novel autoinflammatory syndrome, was described. VEXAS syndrome is attributed to somatic mutations in methionine-41 of *UBA1*, the major E1 enzyme that initiates ubiquitylation. This new disease entity connects seemingly unrelated conditions: systemic inflammatory syndromes (relapsing chondritis, Sweet’s syndrome, and neutrophilic dermatosis) and hematologic disorders (myelodysplastic syndrome or multiple myeloma). Therefore, this article reviews the current literature on both disease entities.

## Introduction

Relapsing polychondritis (RP) is a rare, autoimmune, systemic disease characterized by recurrent episodes of progressive cartilage inflammation that may lead to permanent damage in the affected cartilage. It can occur as an isolated condition (primary RP) or in overlap with other systemic rheumatological diseases. The disease generally follows a fluctuating course, making it difficult to be recognized, otherwise a few cases may develop a progressive course, which can result in significant morbidity and lead to death. The most commonly involved cartilaginous structures include the cartilages of the ears, the hyaline cartilage of the tracheobronchial tree and joints, and the fibrocartilage of the axial skeleton. Immune-mediated damage can spread to involve noncartilaginous tissues that are rich in proteoglycans, such as those in the eyes, inner ear, heart, blood vessels, and kidneys. The RP symptoms of each patient will correspond to the affected site.

RP was described by Rudolf Jaksch von Wartenhorst, an Austrian internist, in 1923 [1]. The patient in the original description was a 32-year-old man who presented with fever, asymmetric polyarthritis, and ear and nose complaints (pain, swelling, and deformity). Nasal cartilage biopsy revealed cartilaginous matrix loss and mucosal hyperplasia. This author classified the patient’s disease as a degenerative disease of the cartilage and named it a polychondropathy. After the initial description, the disease was also called diffuse perichondritis, chondromalacia, chronic atrophic polychondritis, diffuse chondrolysis, and dyschondroplasia. The current name of relapsing polychondritis was introduced by Pearson and colleagues [2] in 1960 to emphasize the episodic course of the disease.

## Epidemiology

Relapsing polychondritis is a rare disease and its prevalence is approximately 4.5 cases per million, affecting all racial groups. However, RP occurs predominantly in Caucasians, and has equal frequency in both sexes. It can occur at any age, with the onset of the disease varying between 20 and 60 years old and with the peak age at onset between 40 and 50 years of age, even though it may occur in any age group. It occurs exceptionally in childhood [3–5]. Over 30% of cases are associated with an existing autoimmune or hematologic comorbidity [6].

### Etiology

The etiology of RP is not known. It is suggested that the disease process may ensue in a genetically predisposed individual on exposure to a triggering factor. The triggering factor may be an infectious agent, chemical or toxin exposure, or direct trauma. Relapsing polychondritis cases have been reported after trauma occurring to the pinna. A possible explanation to this inflammatory reaction to the cartilaginous structures includes cryptogenic antigenic release after trauma and recognition of the antigen by the immune system. Genetic studies have identified HLA-DR4 as the main risk allele for this disease. Familial transmission does not appear to be an etiology of RP [7, 8]. 

### Pathogenesis

The exact mechanism is poorly understood but it is believed to be related to an immune-mediated attack on the proteoglycans of the cartilaginous matrix. The inflammatory infiltrate is composed of mononuclear and plasma cells. In very acute cases, polymorphonuclear cells (PMNs) can be observed within the inflammatory infiltrate.

Cartilage destruction begins from the periphery and shifts toward the center, with breakdown of lacunae and loss of chondrocytes. After chondrocyte apoptosis, focal calcification and cartilage fibrosis occur. Circulating and specific antibodies against collagen types II, IX and XI and matrilin-1, in addition to immune complexes, are observed in patients with RP. Matrilin-1 is a protein from the extracellular matrix of cartilage that is found at high concentrations in the trachea and nasal septum but is absent in articular cartilage. Anti-matrilin-1 antibodies are present in 70% of patients with RP and respiratory symptoms. Mice immunized against matrilin-1 developed respiratory stridor and nasal septal edema, along with erosions in the cartilage and the presence of CD4 + and CD8 + T cells in these lesions. Macrophages release proteolytic enzymes, metalloproteinases (MMP-3), and cathepsin L and K, which lead to cartilage destruction. In addition to humoral immunity, cellular immunity spreads cartilage inflammation. CD4 + cells secrete cytokines such as interleukin 8, macrophage inflammatory protein 1 beta and monocyte activating factor-1, which leads to the recruitment of monocytes and macrophages. Inflammatory mediators activate chondrocytes, PMNs and monocytes, leading to the release of proteolytic enzymes. Immunofluorescence analysis has demonstrated the presence of immunoglobulins and complement at sites of inflammation [9, 10].

### Clinical features

Inflammation of the cartilage is the hallmark of the disease, resulting in recurrent chondritis that can last days to weeks. Some patients may experience constitutional symptoms such as fever, weight loss, and malaise [10].

**Fig. 1:**
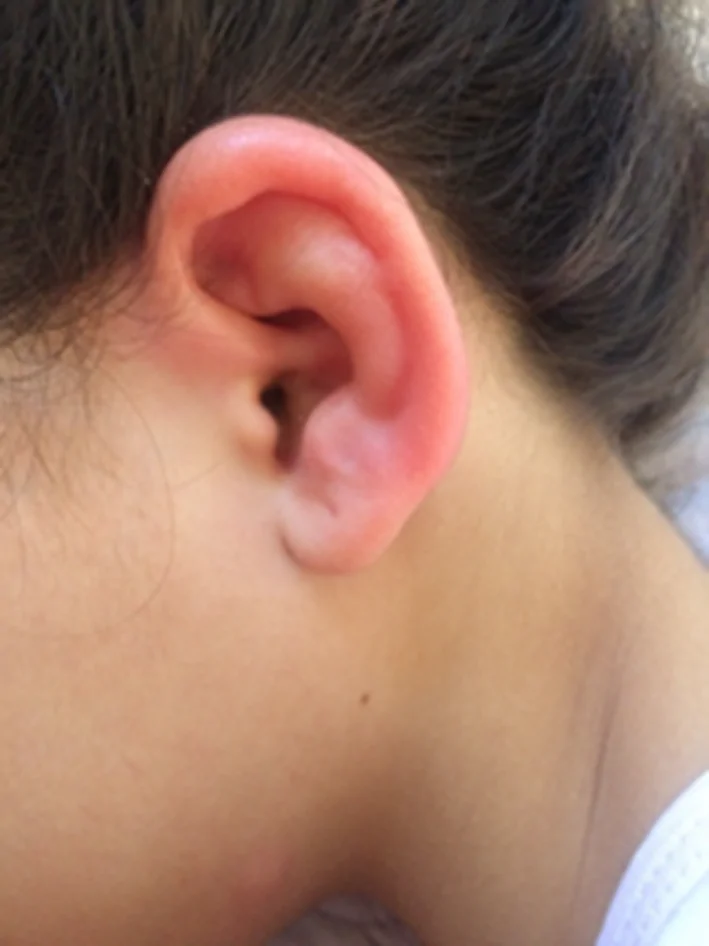
Auricular chondritis (Author’s personal archive)

Involvement of the pinna is present in 20% of patients at the onset of the disease and in 90% during its course. The inflammation spares the ear lobe as it affects only the cartilaginous structure (Fig. 1). After recurrent attacks of inflammation, the pinna becomes flaccid and contorted, resembling the florets of a cauliflower or a fluffy ear.

External auditory canal stenosis, eustachian tube chondritis, and serous otitis media can result in conductive hearing loss. Vasculitis of the internal auditory artery may lead to acute sensorineural hearing loss, with or without vestibular dysfunction [10–11].

**Fig. 2:**
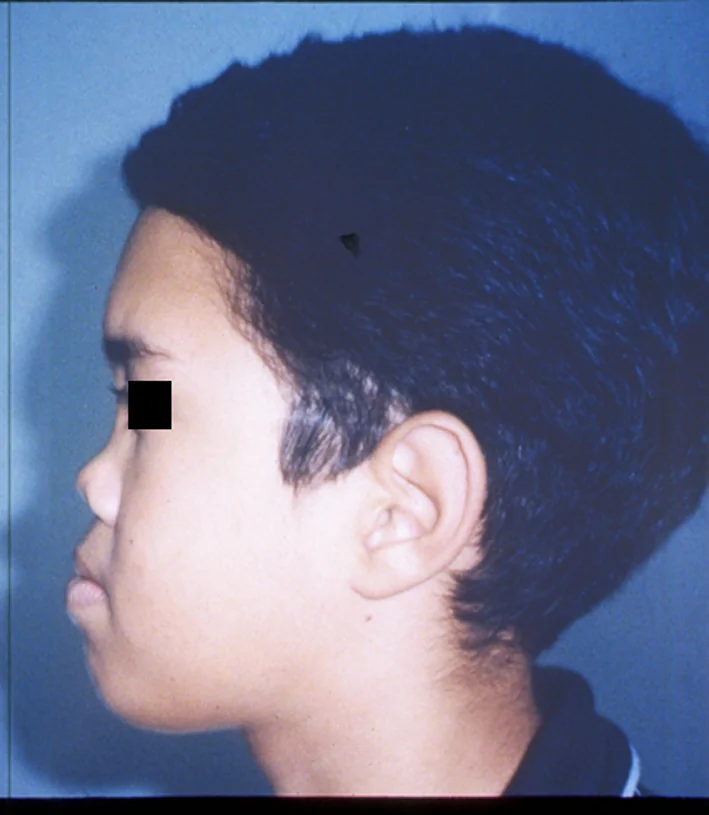
Nasal chondritis (collapse of the nasal bridge causing the “saddle nose” deformity). Author’s personal archive

Chondritis of the nasal cartilage is present in 15% of patients and in up to 70% of the cases as the disease progresses (Fig. 2). It generally has an acute onset with edema, pain, and erythema, affecting the distal cartilaginous portion of the nasal septum. Recurrent episodes can result in the “saddle nose” deformity [10–13].

**Fig. 3:**
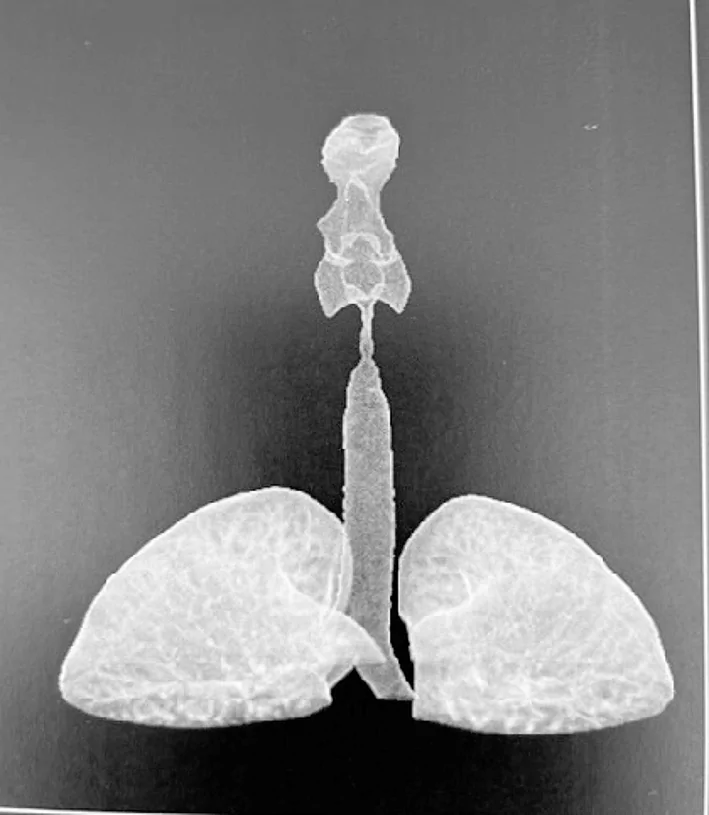
Helicoidal computed tomography showing tracheal stenosis in a patient with RP (Author’s personal archive)

Chondritis of the respiratory tract (e.g., larynx, trachea, bronchial cartilages) affects 10% of patients at the time of their initial presentation and up to 50% during its progression. This manifestation is more commonly observed in women with RP. It is responsible for a third of deaths among individuals with RP and the symptoms of this condition are dysphonia, inspiratory dyspnea, wheezing, and coughing. (Fig. 3) [10–13].

Tracheobronchial involvement may or may not be accompanied by laryngeal chondritis and is potentially the most serious manifestation of RP, causing obstructive respiratory failure or chondromalacia with tracheobronchial tree collapse [10–13]. Additional findings of the tracheobronchial involvement in RP include thickening of the airway wall and luminal narrowing. Costochondritis may occur in 35% of patients with RP.

## Ocular manifestations

**Fig. 4:**
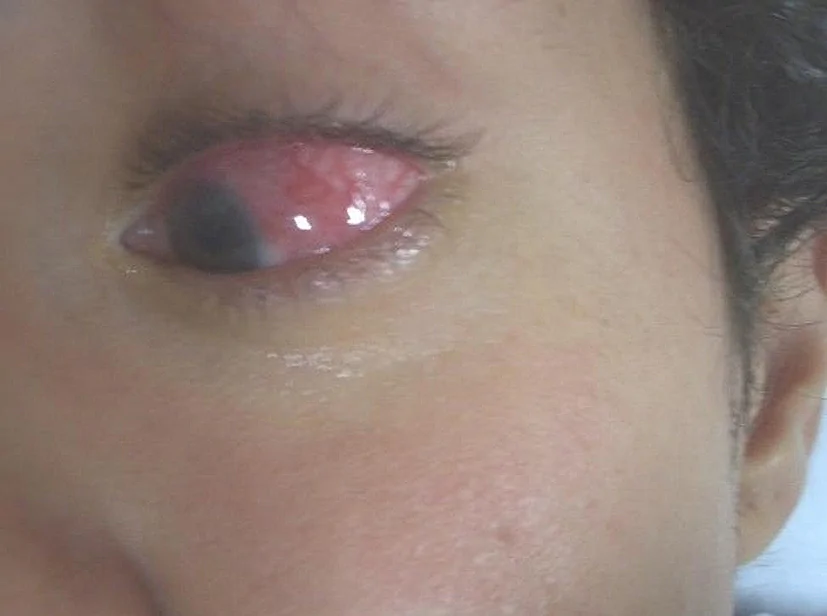
Episcleritis in an adolescent with RP. (Author’s personal archive)

The frequency of ocular involvement is approximately 20% in patients with RP at the disease presentation, reaching up to 65% of patients during follow-up. Episcleritis or scleritis are the most frequent manifestations of the disease and in patients with long-term disease, reactive lymphoid hyperplasia results in a salmon-colored mass in the conjunctiva. Scleritis may be the first manifestation whose study leads to the diagnosis of RP. Scleritis associated with RP is more often bilateral, necrotizing, recurrent and associated with decrease of vision than scleritis associated with other systemic immune-mediated diseases [14, 15]. Recurrent bouts of ocular inflammation can cause thinning of the sclera, allowing a dark or even bluish color of the choroid to be seen through the thinned sclera. Keratoconjunctivitis sicca, keratitis, corneal perforation, iritis, retinopathy and optic neuritis can also occur in RP and may result in blindness. External inflammation of the eyeball can present as an orbital pseudotumor, eyelid edema or extraorbital muscle paralysis. (Fig. 4) [14].

### Musculoskeletal manifestations

Arthralgias and inflammatory arthritis may occur during the course of the disease in 70 to 80% of patients with RP. Serum-negative arthritis is typically asymmetrical, has a migratory pattern and frequently involves the sternoclavicular, costochondral, and manubriosternal joints. Intermittent exacerbations of arthritis are usually observed. Some cases show reduced joint space and osteopenia on X-rays. In severe cases, the clavicles and ribs may dislocate due to cartilage lysis resulting in an unstable chest [12–13].

### Mucocutaneous manifestations

Mucocutaneous changes are present in half of patients with RP. The skin is involved in 20 to 30% of patients with the primary form of RP and in up to 90% of patients with RP associated with myelodysplastic syndrome (MDS). Oral ulcers are the most common RP mucocutaneous manifestations. Oral and genital ulcers associated with cartilaginous inflammation known as MAGIC syndrome (**M**outh **A**nd **G**enital ulcers with **I**nflamed **C**artilage) represent an overlap between Behcet’s syndrome and RP. Other manifestations include erythema nodosum, purpuric lesions, livedo reticularis, urticaria, angioedema, erythema multiforme, and panniculitis. Histological examination of skin biopsies reveals leukocytoclastic vasculitis, thrombosis of cutaneous vessels, septal panniculitis, and neutrophilic dermatosis [13]. The presence of mucocutaneous lesions in a patient with RP should lead to the suspicion of underlying myelodysplasia and VEXAS syndrome, which is an autoinflammatory disease associated with a somatic mutation of the *UBA1* gene [16].

### Hematological manifestations

Hematological manifestations of primary RP are infrequent [12–13]. In the presence of myelodysplastic syndromes in association with polychondritis, especially in men between 60 and 70 years of age, with hematologic abnormalities such as macrocytic anemia, cytopenias, multiple myeloma or monoclonal gammopathy of undetermined significance (MGUS), genotyping of the *UBA1* gene is mandatory to diagnose the recently described VEXAS syndrome. In this condition, vacuoles are observed in myeloid precursor cells in the bone marrow in 100% of reported cases. Venous thrombosis, arterial thrombosis and macrophage activation syndrome may also occur [16].

### Cardiovascular manifestations

**Fig. 5:**
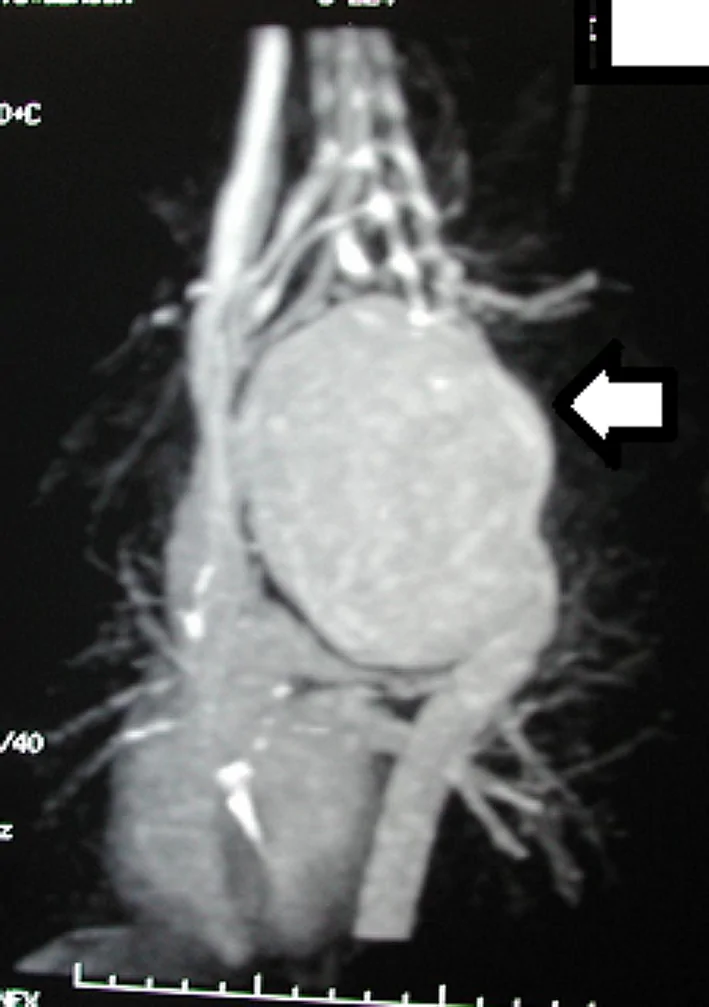
Chest angiotomography showing a thoracic aortic aneurysm in an adolescent with RP. (Author’s personal archive)

The cardiovascular manifestations of RP typically occur in patients with long-standing disease, even when these patients are being treated with immunosuppressive therapy. Aortic insufficiency is the most common cardiovascular complication of RP occurring in 4 to 10% of patients. Mitral regurgitation occurs in 2% of patients with RP. Other manifestations include aortitis, abdominal and thoracic aortic aneurysms with aneurysmal dilation of the aortic arch, conduction abnormalities, pericarditis, as well as arterial thrombosis in case of associated antiphospholipid antibodies [17] (Fig. 5).

### Neurological manifestations

Neurological involvement occurs in less than 3% of patients with RP but can present acutely or subacutely. The cranial nerves are frequently affected by RP, especially the second, sixth, seventh and eighth cranial nerves. The involvement of the second cranial nerve manifests as optic neuritis, while the paralysis of the lateral rectus is a feature of the sixth pair involvement. Paralysis and weakness of the facial nerve indicate the involvement of the seventh and vestibular dysfunction is typically observed when the eighth cranial nerve is affected. Furthermore, headache, seizures, cerebellar dysfunction with ataxia, mental confusion, cerebral aneurysm and aseptic meningitis have also been described in patients with RP [13].

### Renal manifestations

The kidney is an organ rarely affected in RP when it is not associated with other rheumatological diseases. Renal involvement is a sign of poor prognosis in RP. When renal dysfunction occurs, a possible association with systemic lupus erythematosus (SLE) or other systemic diseases should be investigated [13].

### Diagnosis

The diagnosis of RP may be delayed in patients presenting only non-specific symptoms such as fever, weight loss, and fatigue, in the absence of signs of cartilage inflammation.

The diagnosis of RP is primarily based on a combination of clinical features, radiographic findings, and/or biopsy of a cartilaginous site [18]. There is no specific diagnostic test for RP.

Several clinical diagnostic criteria were developed for this disease [18–20]. Hence, tissue biopsy is not always necessary if there is enough clinical evidence.

- McAdam et al. introduced the clinical criteria for RP in 1976 [19].
- These clinical criteria were expanded upon by Damiani et al. in 1979 [20].
- Michet et al. modified the criteria in 1986 [21] (Table 1).

**Table 1:** Box 1: RP diagnostic criteria [19–21]

Authors	Criteria	Conditions required
McAdam et al.	Bilateral auricular chondritis Nasal chondritis Respiratory tract chondritis Non-erosive seronegative polyarthritis Occular inflammation Audiovestibular damage	3 out of 6 criteria
Damiani et al.	Bilateral auricular chondritis (A) Nasal cartilage inflammation (A) Respiratory tract chondritis (A) Non-erosive seronegative polyarthritis (A) Occular inflammation (A) Audiovestibular damage (A) Histologic confirmation (B) Positive response to corticosteroids or dapsone (C)	3 (A) criteria or 1 (A) and (B) or 2 (A) criteria and (C)
Michet et al.	Auricular cartilage inflammation (A) Nasal cartilage inflammation (A) Laryngotracheal cartilage inflammation (A) Ocular inflammation (B) Hearing loss(B) Vestibulary dysfunction (B) Sero-negative arthritis (B)	2 out of 3 (A) criteria or 1 out of 3 (A) criteria and 2 (B) criteria

### Laboratory tests and imaging

There is no specific laboratory test for diagnosing RP. Acute phase reactants may be normal even during disease exacerbation, and abnormal results are nonspecific. Cartilage-specific antibodies are not available in clinical practice and the detection of other autoantibodies (i.e., ANA, RF, ANCA, etc.) is useful for investigating diseases that may be associated with RP. Anti-type II collagen antibody tests are not routinely available, and when these tests are done, not all RP patients present positive results. There are no surrogate markers for ongoing cartilage damage [13].

Pulmonary function tests and bronchoscopy should be indicated on an individual basis. Pulmonary function testing is done to assess airway trapping and lung volumes. Positron Emission tomography with computed tomography (PET-CT) is a new diagnostic modality that seems to be helpful in early disease recognition and in providing a site for targeted biopsy.

Imaging tests such as X-rays, CT scans, and magnetic resonance imaging (MRI) can help evaluate the disease extent and highlight occult airway involvement. An initial screening should be performed in all patients recently diagnosed with RP. PET-CT has proven to be a potentially powerful tool for early diagnosis, especially in patients without organ involvement that is easily accessible for biopsy. This imaging modality also improves the assessment of the extent and disease activity during treatment.

### Differential diagnosis

Chondritis or inflammation of the outer ear can occur due to trauma, such as those that occur in jiu jitsu fighters, or due to infection. In these cases, the chondritis is generally unilateral and also affects the ear lobe. Bilateral auricular chondritis, recurrent and spontaneously resolving, strongly suggests the diagnosis of RP. Other systemic diseases, such as granulomatosis with polyangiitis, rheumatoid arthritis, syphilis, reactive arthritis due to ocular involvement and sarcoidosis, may share some clinical characteristics with RP [13].

### Treatment

Because of its rarity, randomized, placebo-controlled studies have not been conducted in patients with RP. In mild cases of nasal or ear chondritis, non-steroidal anti-inflammatory drugs can be used. If a complete response is not achieved, corticosteroids such as prednisone or equivalent can be used at a dose of 40 to 60 mg/day. In relapsing cases or when other organs are involved, immunosuppressants such as methotrexate, azathioprine, cyclophosphamide, and cyclosporine may be prescribed. Involvement of other organs or systems may require specific interventions (e.g., surgical approach for aneurysm correction, abnormalities in the cardiac conduction system, tracheal stenosis, etc.) [22–25].

The use of biologic agents was evaluated in a multicentre retrospective cohort study in France [22]. This study aimed to evaluate the effectiveness and safety of biologic therapy in RP [22]. Forty-one patients exposed to 105 biologics for 1 year were analyzed. The biologics included TNF inhibitors, tocilizumab, anakinra, rituximab, and abatacept. The overall response rate during the first six months of exposure was 63%, but during this period they were used in combination with corticosteroids. The complete response rate to therapy was 19%. There was a trend toward a higher response rate in patients concomitantly exposed to nonbiologic disease-modifying antirheumatic drugs [22–25].

For patients with severe disease manifestations, other agents are used including JAK inhibitors [26].

### VEXAS syndrome

Within the spectrum of polychondritis, a new syndrome was recently identified in which patients develop inflammatory and hematological symptoms. This new syndrome was named VEXAS syndrome and it is caused by a somatic mutation in the *UBA1* gene in blood cells and appears late in life (i.e., above the age of 50 years). It is an X-linked disease and has therefore been primarily observed in men [27, 28].

VEXAS is often diagnosed as relapsing polychondritis refractory to treatment and it can remain misdiagnosed for many years. The detection of *UBA1* gene mutations in patients with RP ranges from 7.6 to 72.7% [33].

VEXAS is an acronym for **V**acuoles, **E**1 enzyme, **X**-linked, **A**utoinflammatory, **S**omatic [27].

**V** - vacuoles are often seen in cells identified in bone marrow aspirates from patients with VEXAS syndrome.

**E** - **E1** ubiquitin-activating enzyme is encoded by the *UBA1* gene, which is mutated in patients.

**X -** the *UBA1* gene is located on the **X** chromosome.

**A** - patients have **a**utoinflammation.

**S** - the mutations are **s**omatic, meaning they are acquired at some point in life and are not inherited.

### Pathogenesis

VEXAS is an auto-inflammatory disease, identified by a somatic mutation in a gene that encodes E1 enzymes, located on the X chromosome. All patients diagnosed with this syndrome presented cytoplasmic vacuoles, predominantly located in promyelocytes, myelocytes, erythroid precursors and blasts in the bone marrow [27].

The first case was described in 2020, in a man with an inflammatory disease associated with myelodysplasia. In genetic analysis, a mutation in the *UBA1* gene was identified.

The *UBA1* gene is located on the X chromosome and encodes the E1 enzyme, which is the main enzyme that initiates ubiquitination. Ubiquitin is a protein found in eukaryotic cells comprising 76 amino acids, this protein plays an important role in protein regulation, marking unwanted proteins so that they are degraded by a multiprotein complex called the proteasome.

Monocytes carrying *UBA1* variants (mutants) showed decreased ubiquitination and activated innate immune pathways, causing systemic inflammation [27–29].

Mutation in the *UBA1* gene occurs in somatic cells, producing systemic inflammation. In this sense, VEXAS has been called “haemato-inflammatory disease”. This condition manifests as a premalignant condition as it results in myeloproliferation, myelodysplasia, lymphoproliferation, or cellular malignant transformation potential [29].

Somatic mutations in *UBA1* originate in bone marrow stem cells, affecting methionine-41 in the *UBA1* gene, and are restricted to the myeloid cell lineage in peripheral blood. This change gives rise to a complex inflammatory syndrome that manifests itself in adulthood [30].

Sequencing of isolated cell populations revealed that *UBA1* variants were found in more than half of progenitor and myeloid lineage cells, but were absent in T cells, B cells, and fibroblasts.

### Clinical features

In the initial report, 25 patients were diagnosed with VEXAS syndrome based on the confirmation of somatic mutations in codon 41 of *UBA1*. The median age at disease onset was 64 years, and all patients were male. Auricular and/or nasal chondritis was one of the most common organ manifestations, with 15 (60%) patients meeting the classification criteria for RP. According to other cohort studies, the incidence of chondritis is 36–50% [11].

The typical presentation of VEXAS includes fever and systemic features resulting from inflammation, that involves the skin (leukocytoclastic vasculitis and neutrophilic dermatoses), lungs (alveolitis or exudative serositis), cartilage (polychondritis), and blood vessels [12, 27–29].

In general, in its presentation, macrocytosis is identified (mean corpuscular volume > 100 fl.), with progression to myelosuppression characterized by anemia, thrombocytopenia, and lymphopenia. Hematologic disorders are prevalent in VEXAS syndrome, including myelodysplastic syndrome (MDS), multiple myeloma, and monoclonal gammopathy of undetermined significance. Thromboembolic events are also common. The reported incidence of venous thromboembolism (i.e., 36.4%) is much higher than that of arterial thrombosis (1.6%) [42].

VEXAS has a characteristic that differentiates it from most monogenic inflammatory diseases: the identified mutation is present only in cells of somatic origin and not in germ cells [28].

Although the somatic mutations in *UBA1* confirmed the diagnosis of all patients, it is not a widely available test, much less routinely applied.

A fact that draws attention is that patients with VEXAS syndrome meet the criteria for several other conditions, such as RP, polyarteritis nodosa, SLE, and Multiple Myeloma. Chondritis of the ears and nose is a manifestation of VEXAS and up to 8 to 14% of patients with RP may truly have VEXAS as an underlying diagnosis. The clinical spectrum of presentation is still expanding as it is a disease described very recently [16].

Patients with VEXAS may develop a wide range of inflammatory symptoms affecting multiple organs [29, 32–33]. A summary of clinical manifestations is presented in Table 2.

**Table 2:** Summary of clinical characteristics of VEXAS syndrome

Organ	Inflammatory symptoms
Constitutional	Fever Fatigue Weight loss Night sweats
Mucocutaneous	Panniculitis-like nodular lesions Urticarial vasculitis Livedo reticularis Erythema nodosum Neutrophilic dermatosis
Cartilage	Ear and nose chondritis
Lung	Cough and shortness of breath Pulmonary infiltrates Ground-glass opacities Organizing pneumonia Pleural effusion
Musculoskeletal	Arthralgia Arthritis
Vessels	Vasculitis Thrombotic manifestations (Pulmonary embolism, deep venous thrombosis)
Liver/spleen	Hepatosplenomegaly
Heart	Myocarditis
Ophthalmologic	Episcleritis Periorbital edema
Lymphoproliferation	Lymphadenopathy
Other	Macrophage Activation Syndrome [30, 31] Optic neuritis Vestibular dysfunction

Cutaneous manifestations are present in 90% of the patients whose cases have been described.

Acute phase reactant levels are increased in most patients with RP.

On many occasions, patients with VEXAS have an associated disease, including RP, polyarteritis nodosa, Sweet’s syndrome and myelodysplastic syndrome.

The presence of cytoplasmic vacuolation, characteristic of the disease, constitutes a useful marker for its identification and exclusion from differential diagnoses.

Currently, the diagnosis of this syndrome depends solely on the presence of *UBA1* mutations confirmed by Sanger sequencing or next-generation sequencing (NGS), including whole-genome sequencing and whole-exome sequencing, that may favorably serve in the faster and more accurate diagnosis [32, 33].

### Treatment

The optimal treatment of VEXAS is still unknown and patients present a variable response. As it is a relatively new disease, effective therapies still need to be further investigated. Symptoms tend to be refractory, and high-dose corticosteroid therapy appears only temporarily effective and with considerable toxicity.

Immunosuppressants (e.g., Cyclophosphamide, Methotrexate, Mycophenolate mofetil and Azathioprine) and biologic agents (e.g., anti-IL6 receptor tocilizumab) [34, 37], anti-IL-17 (e.g., secukinumab)[35], abatacept [36], anti-IL1 inhibitors [37] (e.g., anakinra, canakinumab), TNF inhibitors [37] (e.g., infliximab and adalimumab), anti-CD20 (e.g., rituximab), and anti-IL-12/IL-23 (e.g., ustekinumab) therapies have been administered to patients with VEXAS syndrome, as well as inhibitors of JAK kinases [38] (e.g., ruxolitinib, tofacitinib and baricitinib), whose efficacy and safety still need to be studied. Given that multiple cytokines are involved in the disease mechanism of VEXAS syndrome, the use of JAK inhibitors seems better therapeutic strategy rather than single cytokine blockade.

One study evaluated the use of azacytidine [39] (a DNA hypomethylating agent), and observed a reduction in inflammatory symptoms for approximately 22 months, but without effectiveness in myelodysplasia [39]. Considering the high prevalence of MDS in VEXAS syndrome, azacytidine could be a good candidate for this syndrome.

If the above-mentioned therapies fail, allogeneic hematopoietic stem cell transplantation (ASCT) may be the last treatment option for VEXAS syndrome and a series of cases showing successful treatment with ASCT has been accumulated [40–42].

Patients still have high mortality due to the high refractoriness rate.

At this time, management must be individualized, based on each patient’s tolerability and response, and on the combined experience of the rheumatologist and hematologist following the patient.

## Conclusion

The finding of polychondritis in a patient can be considered the starting point for the investigation of a series of rheumatic diseases that include classic RP or in association with other conditions, such as rheumatoid arthritis and SLE, as it can also represent a manifestation of this new autoinflammatory disease known as VEXAS.

The occurrence of an inflammatory disease refractory to treatment and associated with hematological abnormalities, the presence of vasculitis, relapsing polychondritis and neutrophilic dermatosis should guide the investigation of VEXAS, especially if the patient is a man over 50 years of age.

## Data Availability

Data sharing not applicable– no new data were generated.
